# Opportunistic Screening Using Low‐Dose CT and the Prevalence of Osteoporosis in China: A Nationwide, Multicenter Study

**DOI:** 10.1002/jbmr.4187

**Published:** 2020-11-04

**Authors:** Xiaoguang Cheng, Kaiping Zhao, Xiaojuan Zha, Xia Du, Yongli Li, Shuang Chen, Yan Wu, Shaolin Li, Yong Lu, Yuqin Zhang, Xigang Xiao, YueHua Li, Xiao Ma, Xiangyang Gong, Wei Chen, Yingying Yang, Jun Jiao, Bairu Chen, Yinru Lv, Jianbo Gao, GuoBin Hong, Yaling Pan, Yan Yan, Huijuan Qi, Limei Ran, Jian Zhai, Ling Wang, Kai Li, Haihong Fu, Jing Wu, Shiwei Liu, Glen M Blake, Perry J Pickhardt, Yuanzheng Ma, Xiaoxia Fu, Shengyong Dong, Qiang Zeng, Zhiping Guo, Karen Hind, Klaus Engelke, Wei Tian, For the China Health Big Data (China Biobank) project investigators

**Affiliations:** ^1^ Department of Radiology Beijing Jishuitan Hospital Beijing China; ^2^ Department of Medical Record Management and Statistics Beijing Jishuitan Hospital Beijing China; ^3^ Department of Health Center Yijishan Hospital of Wannan Medical College Wuhu China; ^4^ Department of Radiology The Affiliated Hospital of Guiyang Medical University Guiyang China; ^5^ Department of Health Management, Henan Provincial People's Hospital People's Hospital of Zhengzhou University Zhengzhou China; ^6^ Department of Radiology The Affiliated Huashan Hospital of Fudan University Shanghai China; ^7^ Department of Radiology The First Affiliated Hospital of Zhengzhou University Zhengzhou China; ^8^ Department of Radiology The Fifth Affiliated Hospital of Sun Yat‐Sen University Zhuhai China; ^9^ Department of Radiology, Ruijin Hospital Shanghai Jiao Tong University School of Medicine Shanghai China; ^10^ Department of Radiology Ningbo Medical Center Li Huili Hospital Ningbo China; ^11^ Department of CT The First Affiliated Hospital of Harbin Medical University Harbin China; ^12^ Institute of Diagnostic and Interventional Radiology Shanghai Jiao Tong University Affiliated Sixth People's Hospital Shanghai China; ^13^ Department of Health Management China‐Japan Friendship Hospital Beijing China; ^14^ Department of Radiology the People's Hospital of Zhejiang Province Hangzhou China; ^15^ Department of Radiology Southwest Hospital, Army Medical University Chongqing China; ^16^ Department of Healthmanagement The Affiliated Hospital of Guiyang Medical University Guiyang China; ^17^ Department of Radiology Yijishan Hospital of Wannan Medical College Wuhu China; ^18^ Department of Radiology Beijing PUMC Hospital Beijing China; ^19^ National Center for Chronic and Noncommunicable Disease Control and Prevention Chinese Center for Disease Control and Prevention Beijing China; ^20^ School of Biomedical Engineering & Imaging Sciences, King's College London St Thomas' Hospital London UK; ^21^ Department of Radiology University of Wisconsin School of Medicine and Public Health Madison WI USA; ^22^ Orthopedics Department The 8th Medical Center of Chinese PLA General Hospital Beijing China; ^23^ Editorial Office of the Chinese Health Management Journal Beijing China; ^24^ Health Management Institute Chinese People's Liberation Army General Hospital Beijing China; ^25^ Department of Radiology Orthopedic Institute of Henan Province Zhengzhou China; ^26^ Department of Sport and Exercise Sciences Durham University Durham UK; ^27^ Department of Medicine 3 FAU University Erlangen‐Nürnberg and Universitätsklinikum Erlangen Erlangen Germany; ^28^ Department of Spine Surgery Beijing Jishuitan Hospital Beijing China

**Keywords:** OPPORTUNISTIC SCREENING, LOW‐DOSE CT, BONE MINERAL DENSITY, OSTEOPOROSIS, PREVALENCE

## Abstract

Opportunistic screening for osteoporosis can be performed using low‐dose computed tomography (LDCT) imaging obtained for other clinical indications. In this study we explored the CT‐derived bone mineral density (BMD) and prevalence of osteoporosis from thoracic LDCT in a large population cohort of Chinese men and women. A total of 69,095 adults (40,733 men and 28,362 women) received a thoracic LDCT scan for the purpose of lung cancer screening between 2018 and 2019, and data were obtained for analysis from the China Biobank Project, a prospective nationwide multicenter population study. Lumbar spine (L_1_–L_2_) trabecular volumetric bone mineral density (vBMD) was derived from these scans using quantitative computed tomography (QCT) software and the American College of Radiology QCT diagnostic criteria for osteoporosis were applied. Geographic regional differences in the prevalence of osteoporosis were assessed and the age‐standardized, population prevalence of osteoporosis in Chinese men and women was estimated from the 2010 China census. The prevalence of osteoporosis by QCT for the Chinese population aged >50 years was 29.0% for women and 13.5% for men, equating to 49.0 million and 22.8 million, respectively. In women, this rate is comparable to estimates from dual‐energy X‐ray absorptiometry (DXA), but in men, the prevalence is double. Prevalence varied geographically across China, with higher rates in the southwest and lower rates in the northeast. Trabecular vBMD decreased with age in both men and women. Women had higher peak trabecular vBMD (185.4 mg/cm^3^) than men (176.6 mg/cm^3^) at age 30 to 34 years, but older women had lower trabecular vBMD (62.4 mg/cm^3^) than men (92.1 mg/cm^3^) at age 80 years. We show that LDCT‐based opportunistic screening could identify large numbers of patients with low lumbar vBMD, and that future cohort studies are now required to evaluate the clinical utility of such screening in terms of fracture prevention and supporting national health economic analyses. © 2020 The Authors. *Journal of Bone and Mineral Research* published by Wiley Periodicals LLC on behalf of American Society for Bone and Mineral Research (ASBMR)..

## Introduction

The prevalence of osteoporosis and the incidence of fragility fracture in China have increased markedly over the last three decades.^(^ ^1^ ^)^ Recent data report an osteoporosis prevalence of 29.1% in women and 6.5% in men aged >50 years, equating to an estimated population prevalence of 49.3 million and 10.9 million, respectively.^(^ ^2^ ^)^ It is estimated that by 2050, there will be 5.99 (95% CI, 5.44 to 6.55) million fractures annually in China, costing $25.43 (95% CI, $23.92 to $26.95) billion, reflecting a 2.7‐fold increase since 2010.^(^ ^3^ ^)^ The increase in osteoporosis and fracture rates reflect in part the rapidly aging population of China^(^ ^4^ ^)^ and therefore reliable estimates of the prevalence of osteoporosis and fracture incidence will be critical for health policy makers and care providers.

Low‐dose computed tomography (LDCT) scans performed for other indications such as lung cancer screening can be used to assess volumetric bone mineral density (vBMD) and screen for osteoporosis simultaneously with no extra equipment, patient time, or radiation exposure, and at no substantial additional cost. Furthermore, vBMD data can be acquired retrospectively. As such, this method could be applied to expand population screening of osteoporosis, particularly in countries or localities where access to dual‐energy X‐ray absorptiometry (DXA) is limited. According to the 2013 International Osteoporosis Foundation (IOF) Asia Pacific Audit report, access to DXA is limited in China with only 0.46 DXA systems per million inhabitants.^(^ ^5^ ^)^ Conversely, access to CT is markedly higher and costs are comparable.^(^ ^6^ ^)^ For the centers participating in the present study the average price for a DXA examination was 15.7 US dollars (USD) (110 Chinese Yuan Renminbi [RMB]) versus 17.2 USD (120.5 RMB) for QCT. Although DXA‐derived areal BMD (aBMD) is required for osteoporosis diagnosis using the World Health Organization criteria, trabecular volumetric BMD (vBMD) derived from CT can be also used for diagnosis based on thresholds published by the American College of Radiology of 120 mg/cm^3^ and 80 mg/cm^3^ to define osteopenia and osteoporosis, respectively, thresholds that were subsequently confirmed for the Chinese population.^(^ ^7^, ^8^, ^9^, ^10^ ^)^ Furthermore, vBMD appears to be more strongly related to fracture risk than DXA aBMD measures.^(^ ^11^, ^12^ ^)^

Early screening for lung cancer in China is performed using low‐dose chest CT as part of a new long‐term health strategy—Healthy China 2030, which focuses on the prevention of disease.^(^ ^13^, ^14^, ^15^ ^)^ We have previously shown that LDCT can be utilized in an opportunistic approach to also measure trabecular vBMD of the lumbar spine with high precision.^(^ ^16^ ^)^ The aim of this population‐based study was to determine the prevalence of osteoporosis in China based on the analysis of lumbar spine vBMD derived from LDCT chest scans obtained for lung cancer screening.

## Subjects and Methods

### Study design

The China Biobank Study is a prospective, nationwide multicenter population cohort study; the study design and protocol have been described elsewhere.^(^ ^17^ ^)^ The program was registered with the US clinical trials database (https://clinicaltrials.gov/ct2/show/NCT03699228; trial identifier: NCT03699228; China Nationwide Multi Center Big Data Study on the Quantitative Computed Tomography [QCT] and Health Status of Check‐Up Population). In the current study, LDCT chest scan data were obtained to retrospectively assess lumbar spine trabecular vBMD. The LDCT chest scans had been obtained primarily for the purpose of lung cancer screening.^(^ ^17^ ^)^ The study was reviewed and approved by the research ethics committee of Beijing Jishuitan Hospital and all participants provided signed informed consent.

### Population cohort

**Fig 1:**
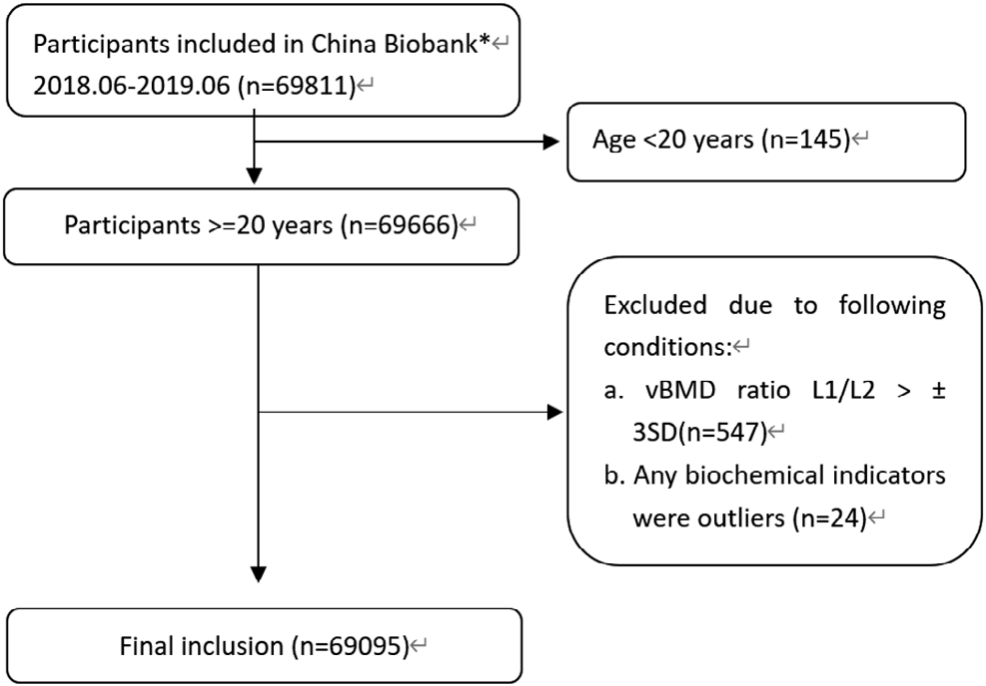
Flowchart of participants. *Data from 13 centers.

China Biobank data were provided for 69,811 participants who had received low‐dose chest CT scans between June 2018 and June 2019 at one of the 13 institutions participating in the China Biobank Study. Data were excluded from the analysis for participants who were aged <20 years or if the vBMD L_1_–L_2_ ratio was outside ±3 standard deviations (SDs). The final sample comprised of 69,095 participants (Fig. 1). There were 40,733 men (mean ± SD, age 49.7 ± 12.7 years; median, 49 years; range, 20 to 98 years) and 28,362 women (age 49.6 ± 12.4 years; median, 49 years; range, 20 to 95 years). Average body mass index (BMI) for men was 25.0 ± 3.1 kg/m^2^ and for women 23.1 ± 3.2 kg/m^2^. Median BMI for men was 24.8 kg/m^2^ (range, 13.3 to 59.4 kg/m^2^) and for women 22.7 kg/m^2^ (range, 14.3 to 45.2 kg/m^2^). The distribution of participants by geographic region was as follows: Northeast China: 6.81%; North China: 3.34%; East China: 47.65%; South China: 5.69%; Central China: 18.13%; and Southwest China: 18.38%. The participants in the present study were in a routine health checkup program^(^ ^17^ ^)^ similar to the subjects in our previously published DXA study.^(^ ^2^ ^)^ Both cohorts were generally healthy subjects with the same recruitment strategy and clinical setting, similar age range, and similar ratio of men/women. Because of the differing availability of DXA and QCT, the participating centers in the two studies were not the same, so the numbers of subjects from different regions of China were not matched. A detailed comparison of the QCT and DXA cohorts is shown in Supplemental Table 1.

### LDCT

A low‐dose chest CT was part of participant's general health checkup protocol; the LDCT was performed according to the same protocol at every center. Mindways QCT Pro (Mindways Software, Inc., Austin, TX, USA) was used for all QCT vBMD measurements and all CT scans were acquired at 120 kVp. The LDCT images were transferred to a QCT workstation for analysis. No extra radiation was involved in this analysis.

### vBMD

**Fig 2:**
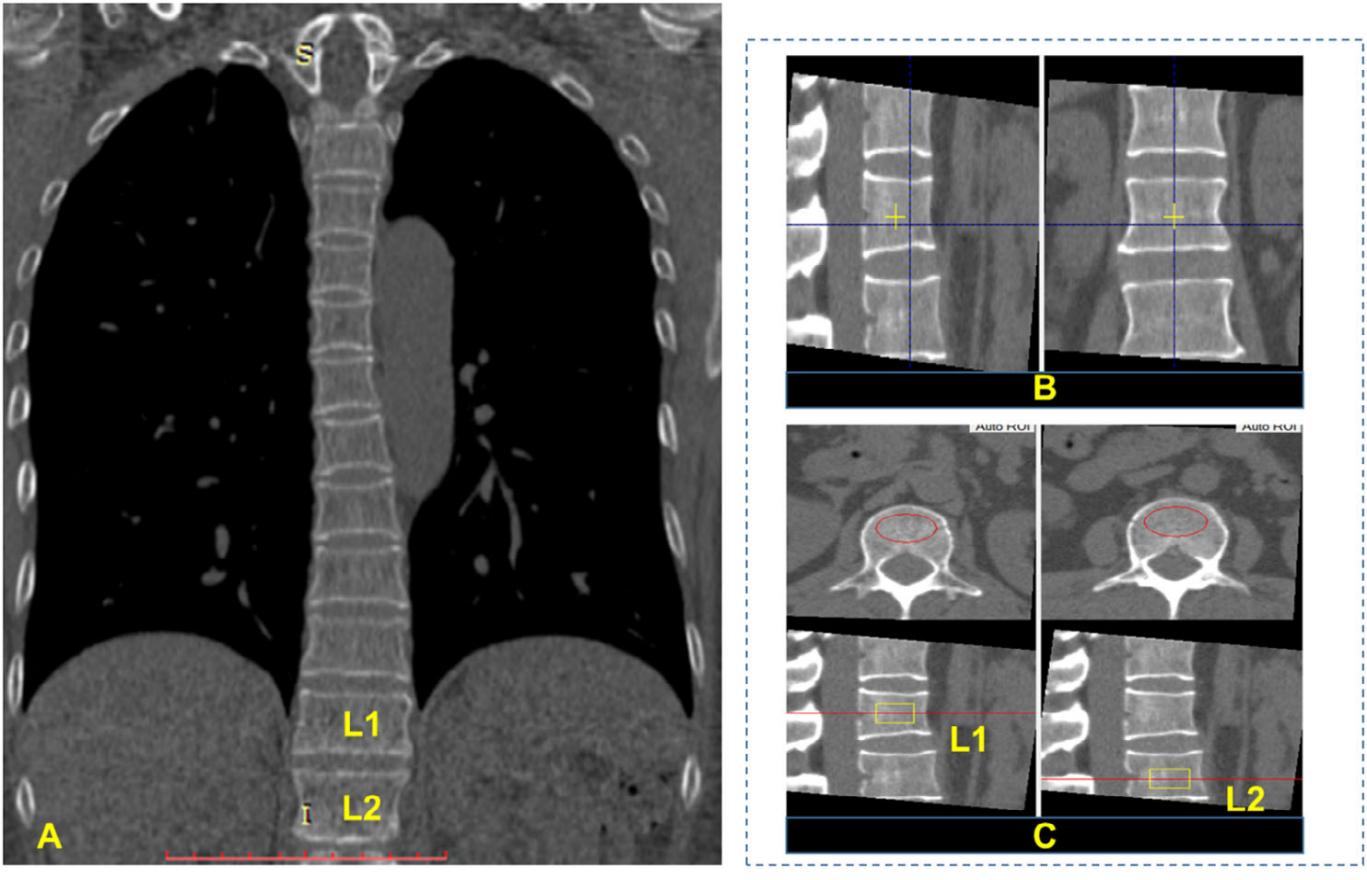
Measurement of vBMD of L1 and L2 with Mindways QCT Pro system. (*A*) Coronal view of a chest LDCT scan. (*B*) Positioning of sagittal and axial views for subsequent automatic placement of analysis VOIs. (*C*) Analysis VOIs shown as red ellipse in axial view and yellow rectangle in sagittal view. LDCT = low‐dose CT; VOI = volume of interest.

Asynchronous BMD calibration in combination with the QCT Pro analysis software (Mindways Software, Inc.) was used to obtain lumbar spine (L_1_–L_2_) trabecular vBMD (mg/cm^3^) (Fig. 2). All analyses were performed by radiologists who were trained and experienced in using the QCT software. Because this protocol involved the post‐imaging processing of existing plain LDCT data, no additional radiation dose was involved. The prevalence of osteoporosis and of low bone mass were defined according to the American College of Radiology QCT diagnostic criteria of vBMD <80 mg/cm^3^ and 80 to 120 mg/cm^3^, respectively.^(^ ^8^, ^9^ ^)^ A validation study has been published confirming the suitability of these criteria in a Chinese population.^(^ ^10^ ^)^

Quality control was ensured throughout the study duration using daily calibration and cross calibration between systems using the European spine phantom (ESP‐145). The quality assurance (QA) results showed the ESP vBMD measured at each center differed by less than 5 mg/cm^3^ on average. Therefore, the original vBMD was used for further analysis. Based on 10 repeated scans of the ESP at each participating center the median coefficient of variation (%CV) for the L_1_–L_3_ ESP vBMD was 0.48% (range, 0.31% to 1.20%).

### Statistical analysis

Participants were stratified by sex, age, BMI, and geographic region. Age groups were created according to 5‐year increments from age 20 to 80+ years. Data were normally distributed and continuous variables were described by mean ± SD. Statistical *t* tests or one‐way analyses of variance (ANOVA) were used for exploring differences between two or more groups. Categorical variables were expressed as frequency and percentage and were analyzed using the chi‐square test. Univariate linear regression models were used to evaluate the effect of demographic parameters on vBMD values. Age groups, BMI levels, geographical region, and the interaction between age and BMI were included in the models. Because the age distribution of the study population differed from that of the Chinese population as a whole, the sex‐specific prevalence of osteoporosis was standardized using the China Biobank study prevalence for each 5‐year age group and the most recent Chinese population data (2010 China Census Data).^(^ ^4^ ^)^ The prevalence of osteoporosis obtained from QCT was compared with published DXA‐derived prevalence rates from 2019.^(^ ^2^ ^)^ All analyses were computed using R version 3.6.0 (R Foundation for Statistical Computing, Vienna, Austria; https://www.r-project.org/).^(^ ^18^ ^)^ Statistical significance was defined by a two‐way test with *p* < .05.

## Results

### BMD and demographic factors

**Fig 3:**
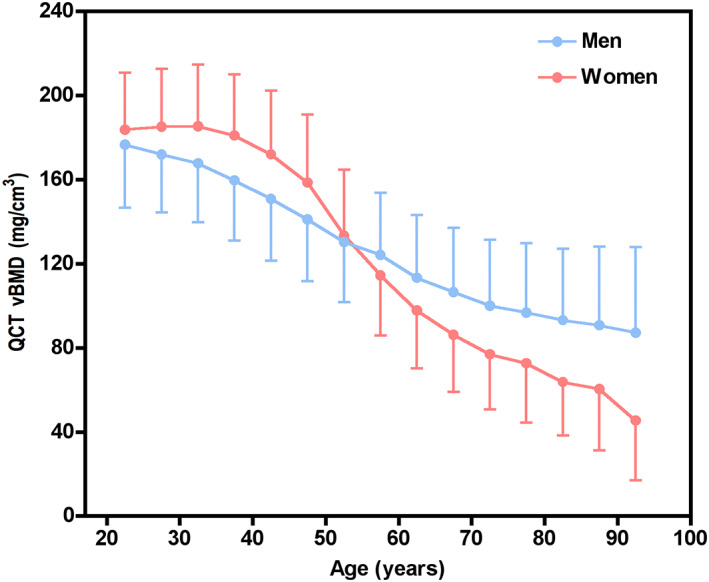
The mean and SD of BMD variation with age. Women had higher vBMD than men before age 50 whereas older women had lower vBMD than men.

The demographic factors of the cohort and the mean vBMD are shown in Table 1. Figure 3 shows the age‐dependent mean vBMD (±1 SD) for each 5‐year interval. Lumbar spine vBMD was highest in the youngest group and decreased progressively with age, varying in women from 185.4 mg/cm^3^ at age 30 to 34 years to 62.4 mg/cm^3^ at age 80+ years, and in men from 176.6 to 92.1 mg/cm^3^ There was a greater rate of bone loss in women than men after the age of 55 years, suggesting the influence of the menopause on bone loss.

**Table 1:** Demographic Characteristics of China Biobank Study Participants

Gender	Age group	*n*	Age (years)	Height (cm)	Weight (kg)	BMI (kg/m^2^)
Men	Total	40733	49.69 ± 12.65	170.36 ± 6.45	72.57 ± 10.58	24.97 ± 3.10
	≥50 years	20154	59.74 ± 8.50	168.99 ± 6.23	71.12 ± 10.01	24.87 ± 2.95
	≥65 years	4828	72.30 ± 6.28	167.19 ± 6.19	68.08 ± 9.89	24.32 ± 3.00
	≥80 years	381	83.69 ± 3.41	165.35 ± 6.22	64.14 ± 9.68	23.43 ± 3.07
Women	Total	28362	49.61 ± 12.37	159.03 ± 5.92	58.36 ± 8.43	23.08 ± 3.15
	≥50 years	13999	59.44 ± 8.07	157.75 ± 5.88	59.26 ± 8.50	23.80 ± 3.08
	≥65 years	3226	71.47 ± 5.88	155.65 ± 5.97	58.57 ± 9.15	24.14 ± 3.30
	≥80 years	729	83.18 ± 2.77	153.19 ± 5.96	55.18 ± 9.67	23.47 ± 3.61

Values for age, height, weight, and BMI are mean ± SD.

### Prevalence of osteoporosis

**Fig 4:**
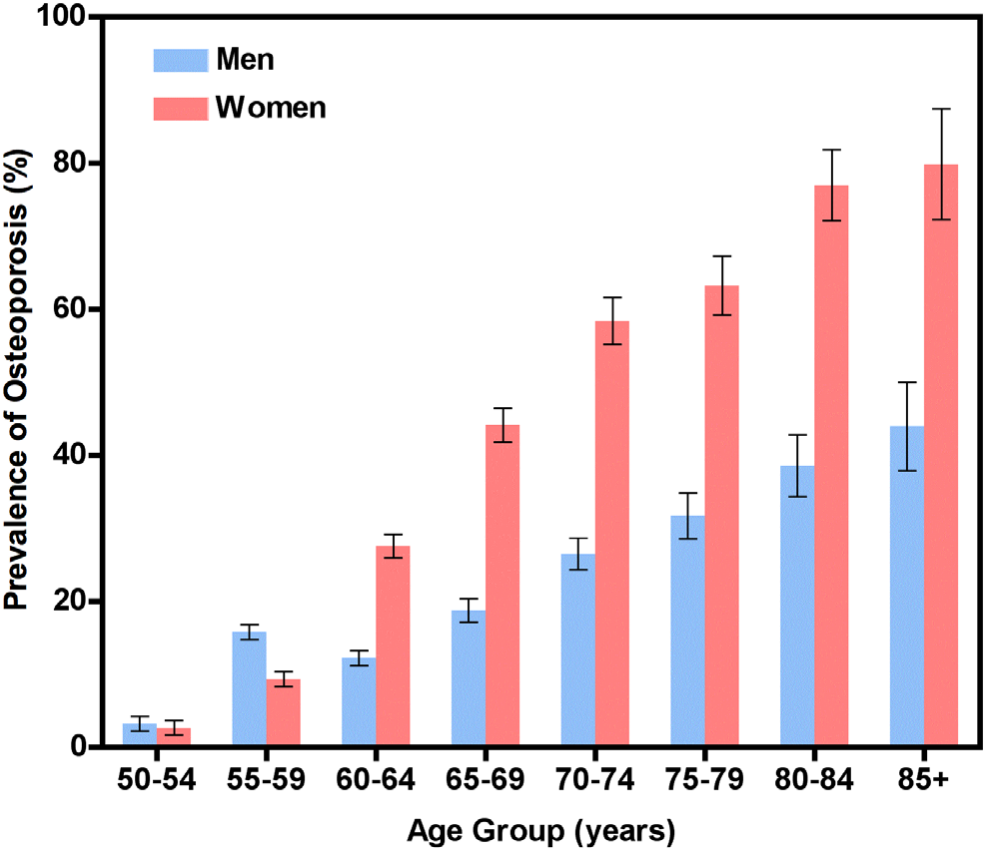
Prevalence of osteoporosis in participants aged 50 years and older. The prevalence increased with age in both men and women. At age 60 years prevalence was already twice as high in women as in men and the ratio increased further at higher ages. Error bars show the 95% confidence intervals.

The prevalence of osteoporosis in participants aged over 50 years is shown in Fig. 4. The percentage of women with osteoporosis increased from 2.8% at age 50 to 54 years to 79.8% at age 85+ years. In men, the prevalence of osteoporosis was 3.2% at age 50 to 54 years and 44.1% at age 85+ years. Following age‐standardization using the 2010 China Census Data,^(^ ^4^ ^)^ the estimated prevalence of osteoporosis for the Chinese population aged over 50 years was 29.0% for women and 13.5% for men, equating to an estimated prevalence of 49.0 million and 22.8 million, respectively (Table 2).

**Table 2:** Age‐Standardized Prevalence of Osteoporosis and Low Bone Mass in the Participants Aged ≥50 Years, Compared With Published DXA Data^(^ ^2^ ^)^

Parameter	Men		Women	
	*n* (%)	Age‐standardized (%)	*n* (%)	Age‐standardized (%)
Current QCT study				
≥50 years (*n*)	20154		13999	
Osteoporosis	2220 (11.02)	13.53	2945 (21.04)	28.99
Low bone mass	8363 (41.50)	42.98	5878 (41.99)	41.07
Published DXA data				
≥50 years (*n*)	21315		20032	
Osteoporosis	920 (4.31)	6.46	4646 (23.19)	29.13
Low bone mass	11264 (52.85)	55.00	10022 (50.03)	49.64

The prevalence of osteoporosis diagnosed by QCT in the current study was compared to recently published prevalence data based on DXA diagnosis.^(^ ^2^ ^)^ The population demographics from the two studies were comparable and participants from both data sets were part of the same general health checkup program. Prevalence rates were calculated using the same methods and age distribution was standardized to the same census data.^(^ ^2^ ^)^ The prevalence of osteoporosis for women was comparable between QCT and DXA, whereas for men the osteoporosis prevalence with QCT was double that of DXA. A more detailed comparison between the two cohorts is given in Supplemental Table 1.

### Regional variation in vBMD and osteoporosis prevalence

**Fig 5:**
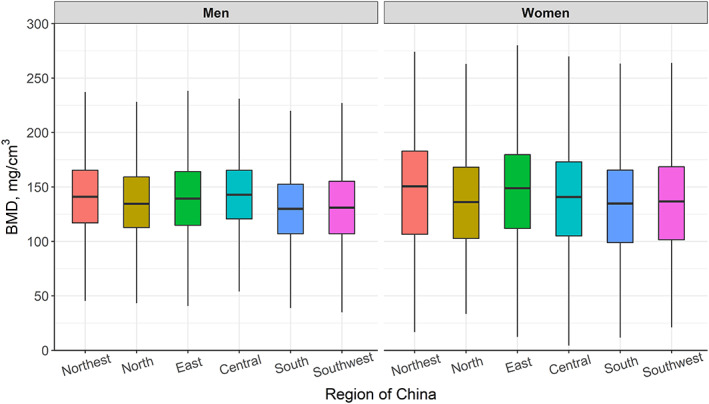
Box and whisker plots showing distributions of vBMD in men and women across different regions of China.

**Fig 6:**
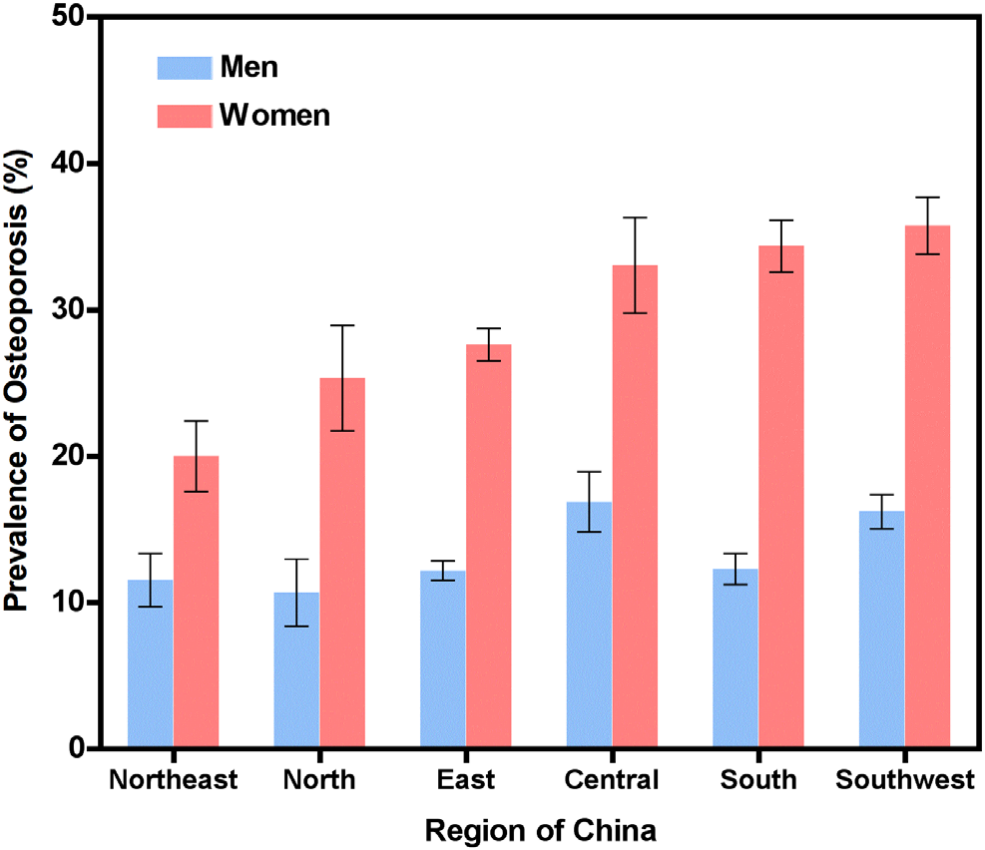
The prevalence of osteoporosis in the ≥50 years group among different regions measured by QCT. Both men and women from Central or Southwest of China had higher prevalence of osteoporosis than those from Northeast or North of China. Error bars show the 95% confidence intervals.

Figure 5 shows the differences in participant vBMD between the different geographic regions of China. ANOVA tests with Bonferroni corrections showed that most differences between regions were statistically significant (Table 3). Figure 6 shows the estimated regional prevalence of osteoporosis in each region for age ≥50 years after standardizing to the age distribution in each region from the 2010 China Census.^(^ ^4^ ^)^ For women, there was a trend for an increase in the prevalence of osteoporosis from north to south China, whereas for men no clear trend was identified. Supplemental Fig. 1 shows regional data from the DXA study^(^ ^2^ ^)^ plotted in a similar way.

**Table 3:** The Values of *p* After Bonferroni Correction of BMD Differences Between Individual Pairs of Geographic Regions

Gender	Region	North China	East China	South China	Central China	Southwest China
Men	Northeast China	.004	1.000	0.800	<.001	<.001
	North China		.024	<.001	<.001	<.001
	East China			.001	<.001	<.001
	South China				<.001	<.001
	Central China					.035
Women	Northeast China	<.001	1.000	.002	<.001	<.001
	North China		<.001	1.000	0.149	1.000
	East China			<.001	<.001	<.001
	South China				<.001	.028
	Central China					0.114

## Discussion

In this large population multicenter study of 69,095 Chinese adults, we show the clinical utility and feasibility of the opportunistic use of low‐dose chest CT scans obtained for lung cancer investigations to identify patients with low lumbar spine vBMD. In doing so, we also report a 29% prevalence of osteoporosis in Chinese women and a 13.5% prevalence in men aged ≥50 years. These age‐standardized estimates are similar to prevalence data from DXA in women, but double that reported in men.^(^ ^2^ ^)^ Furthermore, we report geographical variation in vBMD and osteoporosis for Chinese women. This is the first study to establish Chinese reference data for vBMD using opportunistic screening from low‐dose chest CT in a large population cohort.

The opportunistic screening of osteoporosis using LDCT is clinically feasible and requires no additional exposure to ionizing radiation. This approach is of particular relevance in China because access to CT is greater than access to DXA.^(^ ^6^ ^)^ The value of this approach should also be considered worldwide, given that CT examinations of the thoracic cavity or abdomen are frequently ordered for clinical reasons other than osteoporosis. Thus, in countries where DXA is widely unavailable, there is an unexploited opportunity to use CT scans for the diagnosis of osteoporosis and eventually for fracture risk assessment without any additional radiation exposure to the patient.^(^ ^17^, ^19^, ^20^ ^)^ In the current study, LDCT bone density and osteoporosis assessments were obtained from a large cohort without additional equipment or patient time, suggesting that this approach has potential for opportunistic screening for osteoporosis. However, the clinical utility of such an approach depends on whether lumbar spine vBMD is a sufficiently accurate predictor of future fracture risk to be the basis of treatment decisions. In the United Kingdom, the current clinical guidelines do not recommend DXA aBMD for screening due to a low sensitivity.^(^ ^21^ ^)^ However, there is evidence that vBMD may be a more accurate predictor of fracture risk than aBMD, particularly in men.^(^ ^22^, ^23^ ^)^ Therefore, future cohort studies are warranted to evaluate the clinical utility of opportunistic LDCT screening for fracture prevention and to support national health economic analyses.

This study is the first report of osteoporosis prevalence assessed by QCT in China. In the current study, the prevalence of osteoporosis for women over 50 years was 29.0%, which is comparable to the prevalence of 29.1% reported for diagnoses by DXA.^(^ ^2^ ^)^ However, in men >50 years, the prevalence of osteoporosis by QCT was more than twice as high as by DXA (13.5% versus 6.5%).^(^ ^2^ ^)^ According to these results the ratio of the prevalence rates for osteoporosis in women and men is 2.14 for QCT and 4.46 for DXA. It is important to recognize that the diagnosis of osteoporosis using DXA remains the standard, based on the WHO definition. It should be noted that for the DXA measurements the lowest *T*‐score in three ROIs (L_1_–L_4_, neck, and total hip) was used, whereas for QCT only L_1_–L_2_ vBMD was used. In our earlier studies we found that QCT is more sensitive for detecting osteoporosis than DXA due to the technical superiority of QCT over DXA in men and women.^(^ ^24^, ^25^ ^)^ In the DXA study^(^ ^2^ ^)^ male reference data were used, whereas the International Society for Clinical Densitometry (ISCD) positions suggest the use of female reference data, which would increase the prevalence of osteoporosis in men. The difference in osteoporosis prevalence may reflect a high incidence of degenerative spinal changes in elderly men that can falsely elevate DXA aBMD.^(^ ^24^, ^26^ ^)^

Studies of osteoporosis using the WHO DXA *T*‐score criteria find prevalence in men much lower than that in women. A report of a nationwide population‐based DXA osteoporosis survey with over 20,000 participants conducted by the Chinese Society of Osteoporosis and Bone Mineral Research (CSOBMR) and the Chinese Center for Disease Control and Prevention found the prevalence of osteoporosis for men over 50 years old was 6% compared with 32.1% for women,^(^ ^27^ ^)^ similar to our DXA results. In contrast, an X‐ray survey of osteoporotic fractures of the spine in >14,000 subjects aged 60 to 98 years old conducted in Shanghai reported that the prevalence of vertebral deformity in men >60 years was 17% compared with 17.3% in women.^(^ ^28^ ^)^ The Tromsø study in Norway of 2887 women and men aged 38 to 87 years using DXA Vertebral Fracture Assessment found a slightly higher prevalence of vertebral fractures in men than in women (13.8% for men versus 11.8% for women).^(^ ^29^ ^)^ In contrast, the Dubbo study in Australia reported the residual lifetime fracture risk in a person aged 60 years with average life expectancy was 29% for men and 56% for women based on symptomatic fractures.^(^ ^30^ ^)^ Comparable studies of the incidence of vertebral and hip fracture for the Chinese population are rare. In 2012, Bow and colleagues^(^ ^31^ ^)^ reported that the ratio of clinical fractures in women and men above the age of 65 years was 1.14 for vertebral fractures and 1.98 for hip fractures. From these data, the prevalence of osteoporosis found with QCT seems more comparable to the fracture data. However, further studies are needed to investigate the performance of QCT at estimating fracture risk.

Although there is a diverse literature about age‐related changes and ethnic differences in DXA aBMD, there is relatively little literature for QCT vBMD on these topics. The most commonly cited QCT normative data for a white population was generated from 538 healthy women scanned at the University of California San Francisco (UCSF) in the 1980s using the UCSF liquid calibration standard, data that were subsequently recalibrated to the Imaging Analysis solid phantom (Imaging Analysis, Columbia, KY, USA).^(^ ^32^ ^)^ Men showed a linear decline of vBMD with age, whereas women followed a cubic regression curve with higher peak vBMD than men at around 35 years old, then accelerated bone loss around menopause, leading to lower vBMD than men at older ages. Our data shows that the Chinese population follows a similar variation with age as whites. However, a detailed comparison of vBMD between ethnicities is not possible because the UCSF study was acquired at 80 kVp, whereas the present study was acquired at 120 kVp and calibrated using the Mindways phantom. As in the current study, the Rochester Epidemiology Study found that women aged 20 to 29 years had higher mean lumbar spine trabecular vBMD than men of the same age (203 versus 189 mg/cm^3^),^(^ ^33^ ^)^ and the age‐related trajectory of trabecular bone loss in Japanese women is similar.^(^ ^31^ ^)^ To date, very few studies have reported data from QCT spine imaging for bone density, and have focused primarily on older adults.^(^ ^34^ ^)^

In accordance with previous reports from DXA‐based epidemiological data,^(^ ^2^ ^)^ we found geographical variations in vBMD and the prevalence of osteoporosis in women. The prevalence of osteoporosis was generally greatest and vBMD the lowest in Southern regions of China compared to Northern China, similar to the differences found in our earlier DXA study (Supplemental Fig. 1). Further studies are required to elucidate these geographical variations, but they may be attributable to associations with region‐specific factors including sunlight, climate, food, and lifestyle. Another factor, given the geographical variation in obesity in China,^(^ ^35^ ^)^ may be the effect of body weight on DXA aBMD. Users of GE‐Lunar DXA systems will be familiar with the option to adjust *Z*‐scores for weight. Further studies of geographical variations can inform strategies for allocating resources to the prevention and management of osteoporosis across China.

Low‐dose chest CT scans for early lung cancer screening can save lives.^(^ ^36^ ^)^ The National Lung Screening Trial in 2011 demonstrated that low‐dose chest CT scans are efficacious in reducing lung cancer mortality,^(^ ^37^ ^)^ and LDCT has since been adopted as a clinical standard for health checkup programs in older adults and at risk populations.^(^ ^15^ ^)^ Because the LDCT scans are performed annually, the opportunistic use of these scans for osteoporosis screening offers a clinically and likely economically viable approach to risk identification for targeted fracture prevention initiatives.^(^ ^17^ ^)^

This study has several limitations. First, although the population cohort came from multiple centers, because only the health checkup participants were included the cohort may not be fully representative of the Chinese population with the low‐income population underrepresented. Second, although it is well known that QCT has technical advantages over DXA, because no fracture data were available in this study it is hard to judge whether DXA or QCT correctly diagnose osteoporosis. Third, we did not evaluate risk factors for osteoporosis such as smoking, alcohol consumption, and parental fragility fracture history.

In conclusion, we show the feasibility of identifying large numbers of patients with low lumbar spine vBMD using low‐dose chest CT obtained for other conditions. The age‐standardized prevalence of osteoporosis in patients aged ≥50 years in China using vBMD was 29.0% for women and 13.5% for men. Further studies are required to evaluate the clinical utility of opportunistic screening of the population using of low‐dose chest CT scans for fracture prevention and supporting national health economic analyses.

## Disclosures

Klaus Engelke is a part‐time employee of BioClinica, Inc. Other authors declare no conflict of interest.

## AUTHOR CONTRIBUTIONS

**Xiaoguang Cheng:** Conceptualization; funding acquisition; methodology; writing‐original draft; writing‐review and editing. **Kaiping Zhao:** Data curation; formal analysis; writing‐original draft. **Xiaojuan Zha:** Data curation; formal analysis. **Xia Du:** Resources. **Yongli LI:** Resources. **Shuang Chen:** Resources. **Yan Wu:** Resources. **Shaolin Li:** Resources. **Yong Lu:** Resources. **Yuqin Zhang:** Resources. **Xigang Xiao:** Resources. **YueHua Li:** Resources. **Xiao Ma:** Resources. **Xiangyang Gong:** Resources. **Wei Chen:** Resources. **Yingying Yang:** Resources. **Jun Jiao:** Resources. **Bairu Chen:** Resources. **Yingru Lv:** Resources. **Jianbo Gao:** Resources. **Guobin Hong:** Resources. **Yaling Pan:** Resources. **Yan Yan:** Resources. **Huijuan Qi:** Resources. **Limei Ran:** Resources. **Jian Zhai:** Resources. **Ling Wang:** Conceptualization; project administration; writing‐original draft; writing‐review and editing. **Kai Li:** Conceptualization; project administration; writing‐original draft; writing‐review and editing. **Haihong Fu:** Resources. **Jing Wu:** Resources. **Shiwei Liu:** Resources. **Glen Blake:** Writing‐original draft; writing‐review and editing. **Perry J Pickhardt:** Visualization. **yuanzheng ma:** Resources. **Xiaoxia Fu:** Resources. **Shengyong Dong:** Data curation; visualization. **Qiang Zeng:** Resources. **Zhiping Guo:** Resources. **Karen Hind:** Writing‐original draft; writing‐review and editing. **Klaus Engelke:** Writing‐original draft; writing‐review and editing. **Wei Tian:** Conceptualization; funding acquisition; project administration; resources; supervision; writing‐review and editing.

## Supporting information


**Supplemental Table 1** Comparisons of demographic characteristics between the participants aged 50 years and older from the current QCT study and their counterparts from published DXA data
**Supplemental Fig. 1** The prevalence of osteoporosis in the ≥50 years group among different regions measured by DXA.
