# Supplementary material for: Opportunistic Screening Using Low‐Dose CT and the Prevalence of Osteoporosis in China: A Nationwide, Multicenter Study
Source: J Bone Miner Res. 2020 Nov 4;36(3):427–35. doi: 10.1002/jbmr.4187 (PMC7988599; doi:10.1002/jbmr.4187)
Supplement: Supplementary file 1 — Supplemental Table 1 Comparisons of demographic characteristics between the participants aged 50 years and older from the current QCT study and their counterparts from published DXA data Supplemental Fig. 1 The prevalence of osteoporosis in the ≥50 years group among different regions measured by DXA. [file JBMR-36-427-s001.docx]

**Supplement Section**

**Supplemental Table 1**. Comparisons of demographic characteristics between the participants aged 50 years and older from the current QCT study and their counterparts from published DXA data ^(2)^

|  | Men aged 50 years and older | | | Women aged 50 years and older | | |
| --- | --- | --- | --- | --- | --- | --- |
|  | QCT study | DXA study | P value | QCT study | DXA study | P value |
| Age (years) | 59.7±8.5 | 58.9±7.7 | <0.0001 | 59.4±8.1 | 59.9±8.0 | <0.0001 |
| Height (cm) | 169.0±6.2 | 171.6±5.9 | <0.0001 | 157.8±5.9 | 159.5±5.5 | <0.0001 |
| Weight (kg) | 71.1±10.0 | 75.2±10.4 | <0.0001 | 59.3±8.5 | 62.4±9.0 | <0.0001 |
| BMI (kg/m^2^) | 24.9±2.9 | 25.5±3.0 | <0.0001 | 23.8±3.1 | 24.5±3.3 | <0.0001 |
| Region [N (%)] |  |  |  |  |  |  |
| Northeast China | 1169 (5.8) | 4142(19.4) |  | 1048 (7.5) | 3221(16.1) |  |
| North China | 707 (3.5) | 9697(45.5) |  | 560 (4.0) | 7012(35.0) |  |
| East China | 9453(46.9) | 7227(33.9) |  | 6516(46.6) | 9361(46.7) |  |
| South China | 1272 (6.3) | - |  | 801 (5.7) | - |  |
| Central China | 3677(18.2) | - |  | 2721(19.4) | - |  |
| Southwest China | 3876(19.2) | 249 (1.2) |  | 2353(16.8) | 438 (2.2) |  |


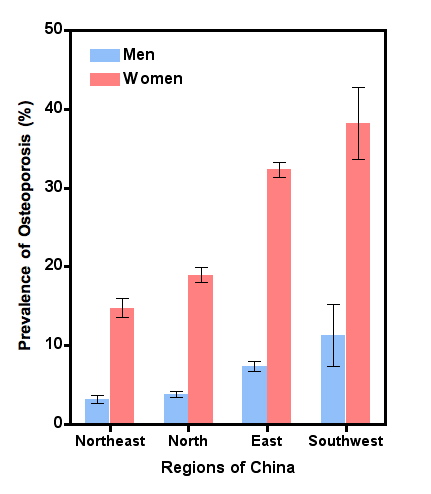


**Supplemental Figure 1**. The prevalence of osteoporosis in the ≥50 years group among different regions measured by DXA ^(2)^. Error bars show the 95% confidence intervals. Regional differences are similar to those determined by QCT shown in Figure 6.
